# Epidemiology of Diphtheria in Yemen, 2017-2018: Surveillance Data Analysis

**DOI:** 10.2196/27590

**Published:** 2021-06-02

**Authors:** Suaad Ameen Moghalles, Basher Ahmed Aboasba, Mohammed Abdullah Alamad, Yousef Saleh Khader

**Affiliations:** 1 Yemen Field Epidemiology Training Programme Ministry of Public Health and Population Sana'a Yemen; 2 Department of Community Medicine, Public Health and Family Medicine Faculty of Medicine Jordan University of Science and Technology Irbid Jordan

**Keywords:** diphtheria, epidemiology, incidence, case fatality rate

## Abstract

**Background:**

As a consequence of war and the collapse of the health system in Yemen, which prevented many people from accessing health facilities to obtain primary health care, vaccination coverage was affected, leading to a deadly diphtheria epidemic at the end of 2017.

**Objective:**

This study aimed to describe the epidemiology of diphtheria in Yemen and determine its incidence and case fatality rate.

**Methods:**

Data were obtained from the diphtheria surveillance program 2017-2018, using case definitions of the World Health Organization. A probable case was defined as a case involving a person having laryngitis, pharyngitis, or tonsillitis and an adherent membrane of the tonsils, pharynx, and/or nose. A confirmed case was defined as a probable case that was laboratory confirmed or linked epidemiologically to a laboratory-confirmed case. Data from the Central Statistical Organization was used to calculate the incidence per 100,000 population. A *P* value <.05 was considered significant.

**Results:**

A total of 2243 cases were reported during the period between July 2017 and August 2018. About 49% (1090/2243, 48.6%) of the cases were males. About 44% (978/2243, 43.6%) of the cases involved children aged 5 to 15 years. Respiratory tract infection was the predominant symptom (2044/2243, 91.1%), followed by pseudomembrane (1822/2243, 81.2%). Based on the vaccination status, the percentages of partially vaccinated, vaccinated, unvaccinated, and unknown status patients were 6.6% (148/2243), 30.8% (690/2243), 48.6% (10902243), and 14.0% (315/2243), respectively. The overall incidence of diphtheria was 8 per 100,000 population. The highest incidence was among the age group <15 years (11 per 100,000 population), and the lowest incidence was among the age group ≥15 years (5 per 100,000 population). The overall case fatality rate among all age groups was 5%, and it was higher (10%) in the age group <5 years. Five governorates that were difficult to access (Raymah, Abyan, Sa'ada, Lahj, and Al Jawf) had a very high case fatality rate (22%).

**Conclusions:**

Diphtheria affected a large number of people in Yemen in 2017-2018. The majority of patients were partially or not vaccinated. Children aged ≤15 years were more affected, with higher fatality among children aged <5 years. Five governorates that were difficult to access had a case fatality rate twice that of the World Health Organization estimate (5%-10%). To control the diphtheria epidemic in Yemen, it is recommended to increase routine vaccination coverage and booster immunizations, increase public health awareness toward diphtheria, and strengthen the surveillance system for early detection and immediate response.

## Introduction

Diphtheria is an acute bacterial disease caused by *Corynebacterium diphtheria* and presents most commonly as a membranous pharyngitis. Symptoms occur after an incubation period of 2 to 5 days [1]. The organism produces a toxin that causes necrosis of the tissues, leading to respiratory obstruction, heart failure, and death. Diphtheria was one of the most common causes of morbidity and mortality among children in the prevaccine era. The mortality rate associated with diphtheria was as high as 50% but dropped to about 15% after widespread use of diphtheria antitoxin treatment [2].

Throughout history, diphtheria has remained one of the most frightening infectious diseases globally, causing overwhelming epidemics with high case fatality rates and mainly affecting children. However, most cases in outbreaks, such as the large outbreak in the Russian Federation in the 1990s [3], and cases reported in the United States since 1980 involved individuals aged 15 years or older [4]. Individuals, particularly children, who are not vaccinated or are partially vaccinated are at risk of diphtheria. Moreover, adults are at high risk as immunity due to vaccination wanes over time [2].

After the introduction of the diphtheria vaccine in the United Kingdom and subsequently worldwide in the 1940s to 1950s [5], diphtheria was practically eliminated, and it became an uncommon disease worldwide. However, diphtheria remains an important health problem in countries with poor routine vaccination coverage. There is global concern that diphtheria is re-emerging. Several outbreaks of diphtheria have been reported from Eastern Europe [6], Southeast Asia, South America [7], and North Africa [8]. In countries of the Eastern Mediterranean Region (EMR), diphtheria continues to occur in the form of localized outbreaks. In 2017, 600 cases of diphtheria were reported in the countries of the EMR, including Islamic Republic of Iran (two cases), Iraq (two cases), Pakistan (560 cases), Sudan (two cases), Qatar (two cases), Afghanistan (one case), Saudi Arabia (one case), and Yemen (30 cases) [9].

In Yemen, the Expanded Program on Immunization (EPI) was established in 1977. The strategy for EPI was to reach 90% coverage at the national level for Penta-3. Although vaccines for major vaccine-preventable diseases are available free for the public under the EPI, vaccine-preventable diseases still cause nearly one-third of the total deaths among Yemeni children under 5 years of age. Before the conflict that began in late 2014, Yemen had a stable vaccination coverage reaching 70% to 80% of the target population; however, this significantly dropped after the war [10]. Vaccine-preventable diseases, such as measles, cholera, and diphtheria, saw a sudden surge after the beginning of the war [10]. Many sporadic outbreaks of diphtheria from different parts of the country were observed. Lately, in October 2017, a diphtheria outbreak occurred in Yareem district in Ibb governorate, and 11 cases were reported with a case fatality rate (CFR) of 27% [11].

The Diphtheria Containment Program was established in February 2018 as a response to the re-emergence of diphtheria in Yemen. Diphtheria mortality- and morbidity-related data are collected daily from health facilities using a case investigation form (Multimedia Appendix 1), which includes information on demographics, clinical signs and symptoms, outcomes, laboratory data, vaccination status, and case management. The recent diphtheria epidemic in Yemen has highlighted the need for diphtheria surveillance data analysis to be used to provide information, such case fatality, vaccination status, age-specific incidence rate, geographical area, and risk groups, for further epidemic preparedness. This study aimed to describe the epidemiology of diphtheria and determine its incidence and CFR.

## Methods

Data of patients with diphtheria were obtained from the diphtheria program in the form of a line list that was collected daily by the electronic Diseases Early Warning System. A probable case was defined as a case involving a person having laryngitis, pharyngitis, or tonsillitis and an adherent membrane of the tonsils, pharynx, and/or nose. A confirmed case was defined as a probable case that was laboratory confirmed or linked epidemiologically to a laboratory-confirmed case [12]. Data were reviewed and cleaned. For calculations of incidence rate, we used the population at risk from the Central Statistics Organization. Surveillance data were analyzed using Epi Info (Centers for Disease Control and Prevention) and Excel 2013 (Microsoft Corp). Data were described using simple descriptive methods, rate calculations, and graphs.

## Results

### Case Characteristics

**Figure 1:**
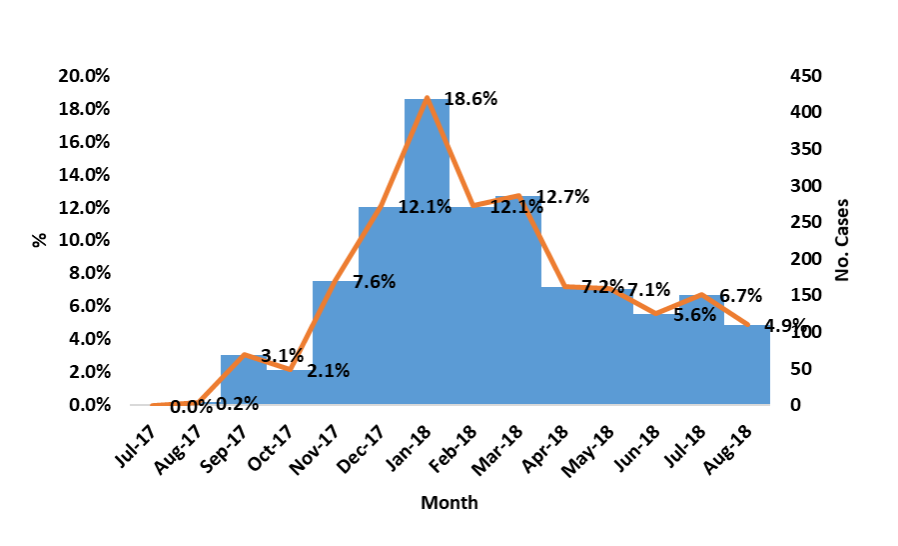
The distribution of 2243 diphtheria cases reported during the period from July 2017 to August 2018 in Yemen.

A total of 2243 cases were reported during the period between July 2017 and August 2018. The number of cases gradually increased from July 2017, with the peak occurring in January 2018, and then decreased until August 2018 (Figure 1). About 49% (1090/2243, 48.6%) of the cases involved males and 51% (1153/2243, 51.4%) involved females. About 44% (978/2243, 43.6%) of the cases involved children aged 5 to 15 years. Diphtheria cases were reported from 215 districts in 20 governorates. Respiratory tract infection was the predominant symptom (2044/2243, 91.1%), followed by pseudomembrane (1822/2243, 81.2%).

### Vaccination Status

Based on the vaccination status, the percentages of partially vaccinated, vaccinated, unvaccinated, and unknown status patients were 6.6% (148/2243), 30.8% (690/2243), 48.6% (10902243), and 14.0% (315/2243), respectively. Zero dose reporting gradually increased by age from 35% in age group <5 years to 74% in age group >45 years, while three doses of the vaccine decreased gradually with age.

### Diphtheria Incidence Rate and the CFR

Table 1 shows the incidence rate of diphtheria per 100,000 population by age group and governorate. Table 2 shows the CFR. The overall incidence of diphtheria was 8 per 100,000 population at affected areas. The highest incidence was among the age group <15 years (11 per 100,000) and the lowest incidence was among the age group ≥15 years (5 per 100,000). The incidence rate varied widely across governorates. The highest incidence rate (20 per 100,000 population) was in Al Dhale’e, Ibb, and Sana’a, while the lowest incidence rate (7 per 100,000 population) was in other (n=17) governorates. The overall CFR among all age groups was 5%, and it was higher (10%) in the age group <5 years. The CFR was higher (22%) in difficult access governorates, including Raymah, Abyan, Lahj, Al Jawf, and Sa’adah.

**Table 1:** Diphtheria incidence by age and governorate in Yemen from 2017 to 2018.

Characteristic		Population (N=28,384,959)	Number of cases (N=2243)	Incidence (per 100,000 population)	*P* value
**Age (years)**					<.001
	<15	12,688,111	1390	11	
	15-35	9,836,674	681	7	
	>35	5,860,174	172	3	
**Governorates**					<.001
	Three governorates^a^	4,880,845	968	20	
	Others (n=17)	23,504,114	1275	7	

^a^Al Dhale’e, Ibb, and Sana’a.

**Table 2:** Diphtheria case fatality rate by age and governorate in Yemen from 2017 to 2018.

Characteristic		Number of cases (N=2243)	Number of deaths (N=120)	Case fatality rate	*P* value
**Age (years) **					<.001
	<5	413	42	10%	
	≥5	1830	78	4%	
**Governorate**					<.001
	Difficult access^a^	92	20	22%	
	Normal access	2151	100	10%	

^b^Raymah, Abyan, Sa'ada, Lahj, and Al Jawf.

### Incidence Rate of Diphtheria by Place

**Figure 2:**
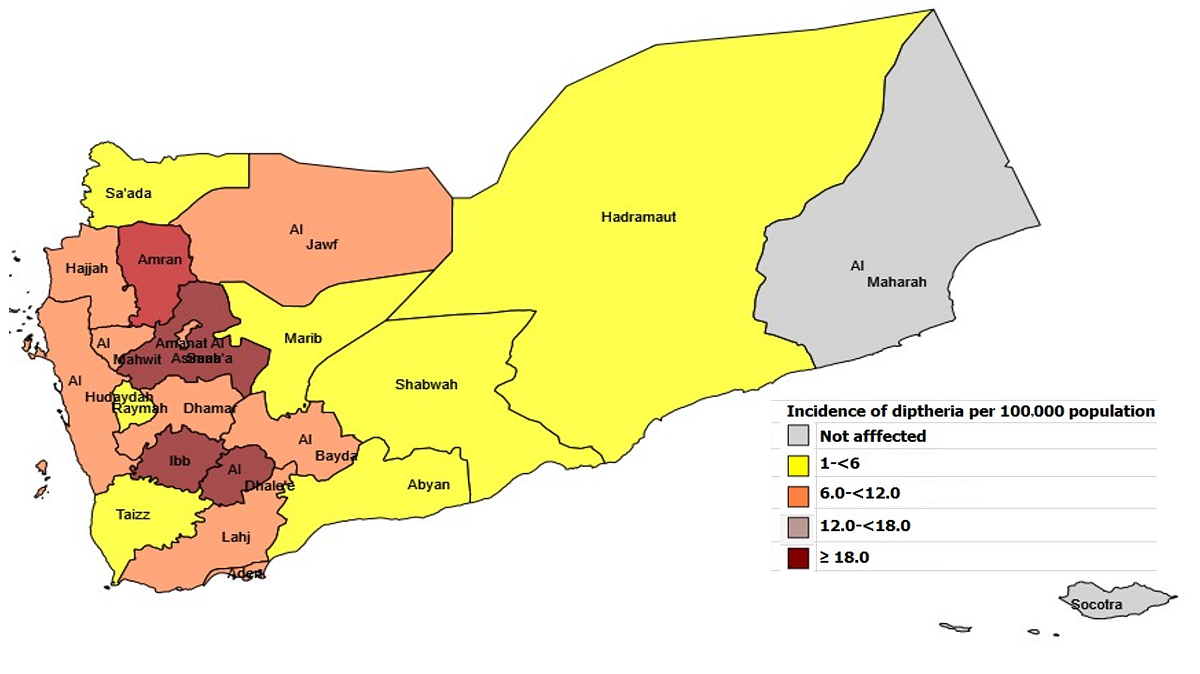
Incidence rate of diphtheria across governorates in Yemen.

Figure 2 provides a heat map showing that the incidence rate of diphtheria varied widely across governorates. The incidence shows that there was an increase in the number of diphtheria cases, with the highest rate of ≥18 per 100,000 population in Al Dhale’e, Ibb, and Sana’a, and the lowest rate of <6 per 100,000 population in Raymah, Shabwah, Taizz, and Abyan (Figure 2).

## Discussion

### Principal Findings

Diphtheria has re-emerged after more than 30 years, and this has led to a huge public health concern in Yemen, which has a poor health system, low coverage of diphtheria vaccination, a low socioeconomic status, and population migration. Diphtheria has re-emerged as a deadly epidemic since the end of 2017. Our findings showed a notable rise in the number of diphtheria cases during the months between October 2017 and January 2018, which are considered winter months. This finding is consistent with the findings of previous studies in Yemen and Indonesia [11,13,14]. A decline in the cases after the peak started in February and continued until August 2018. This decline might be due to control of the spread as a result of vaccination campaigns that were implemented in February and May of 2018.

In terms of age distribution, previous reviews reported a shift in the age group affected by diphtheria to older children and adults [15,16], and this is similar to our finding that the age group 5-15 years was the most affected. However, this finding is not consistent with the findings of a previous study in Yemen [13], which found that children aged <5 years were more affected. This might be related to the immunization status of the population as well as the changing socioeconomic conditions in a given area [16].

Diphtheria outbreaks often occur because of low childhood vaccination, as well as lack of booster vaccinations for older children and adults [17]. This supports our findings that most affected cases were unvaccinated, which is consistent with the findings of previous studies in India and Indonesia reporting that mortality occurred in more than 70% of patients who did not receive complete immunization [14,18]. Our study confirms that raising immunity levels in the population through vaccination remains a critical tool for diphtheria control.

The incidence rate and CFR varied across governorates. This might be due to the variation of reporting. For example, Aden reported cases from all its districts, while Hadaramout reported cases from 7% of its districts. Improving reporting in all governorates will help in providing a complete picture of the epidemiology of diphtheria. The overall CFR was 5%, which is similar to the estimate reported in a previous study from India [19]. This estimation falls in the World Health Organization (WHO) reported range of 5% to 10%. However, five governorates that were difficult to access had a CFR twice that of the WHO estimate.

### Conclusion

Diphtheria affected a large number of people in Yemen in 2017-2018. The majority of patients were partially vaccinated or not vaccinated. Children aged ≤15 years were more affected, with higher fatality among children aged <5 years. Five governorates that were difficult to access had a CFR twice that of the WHO estimate. To control the diphtheria epidemic in Yemen, it is recommended to increase routine vaccination coverage and booster immunizations, and strengthen the surveillance system for early detection and immediate response.

## Appendix

Multimedia Appendix 1 Diphtheria investigation form.

## Abbreviations

CFR: case fatality rate
EMR: Eastern Mediterranean Region
EPI: Expanded Program on Immunization
WHO: World Health Organization

## References

[1] Hamborsky J, Kroger A, Wolfe C, Centers for Disease Control and Prevention (2015). Epidemiology and Prevention of Vaccine-Preventable Diseases.

[2] (2006). Diphtheria vaccine = Vaccin antidiphtérique. World Health Organization.

[3] Vitek C, Wharton M (1998). Diphtheria in the former Soviet Union: reemergence of a pandemic disease. Emerg Infect Dis.

[4] Chen RT, Broome CV, Weinstein RA, Weaver R, Tsai TF (1985). Diphtheria in the United States, 1971-81. Am J Public Health.

[5] Galazka AM, Robertson SE (1995). Diphtheria: Changing patterns in the developing world and the industrialized world. Eur J Epidemiol.

[6] Wagner KS, White JM, Lucenko I, Mercer D, Crowcroft NS, Neal S, Efstratiou A, Diphtheria Surveillance Network (2012). Diphtheria in the postepidemic period, Europe, 2000-2009. Emerg Infect Dis.

[7] Santos LS, Sant'Anna LO, Ramos JN, Ladeira EM, Stavracakis-Peixoto R, Borges LLG, Santos CS, Napoleao F, Camello TCF, Pereira GA, Hirata R, Vieira VV, Cosme LMSS, Sabbadini PS, Mattos-Guaraldi AL (2014). Diphtheria outbreak in Maranhão, Brazil: microbiological, clinical and epidemiological aspects. Epidemiol. Infect.

[8] Besa NC, Coldiron ME, Bakri A, Raji A, Nsuami MJ, Rousseau C, Hurtado N, Porten K (2013). Diphtheria outbreak with high mortality in northeastern Nigeria. Epidemiol. Infect.

[9] Global Health Observatory Data Repository (Eastern Mediterranean Region). World Health Organization.

[10] Qirbi N, Ismail SA (2016). Ongoing threat of a large-scale measles outbreak in Yemen. The Lancet Global Health.

[11] (2017). Diphtheria Outbreak in Yareem District, Ibb governorate, October 2017. Outbreak Investigation.

[12] (1999). WHO-recommended standards for surveillance of selected vaccine preventable diseases. World Health Organization.

[13] Jones E, Kim-Farley RJ, Algunaid M, Parvez A, Ballad A, Hightower W, Orenstein A, Broome V (1985). Diphtheria: a possible foodborne outbreak in Hodeida, Yemen Arab Republic. Bull World Health Organ.

[14] Arguni E, Karyanti MR, Satari HI, Hadinegoro SR (2021). Diphtheria outbreak in Jakarta and Tangerang, Indonesia: Epidemiological and clinical predictor factors for death. PLoS One.

[15] Clarke KEN (2017). Review of the epidemiology of diphtheria - 2000-2016. World Health Organization.

[16] Galazka A (2000). The changing epidemiology of diphtheria in the vaccine era. J Infect Dis.

[17] Bhattacharyya H, Sarkar A, Medhi G, Pala S (2016). Diphtheria outbreak in a district in Meghalaya, India: an overview. Int J Community Med Public Health.

[18] Meera M, Rajarao M (2014). Diphtheria in Andhra Pradesh–a clinical-epidemiological study. Int J Infect Dis.

[19] Nath B, Mahanta T (2010). Investigation of an outbreak of diphtheria in borborooah block of dibrugarh district, assam. Indian J Community Med.

