# Supplementary figures and images for: Epidemiology of Diphtheria in Yemen, 2017-2018: Surveillance Data Analysis
Source: JMIR Public Health Surveill. 2021 Jun 2;7(6):e27590. doi: 10.2196/27590 (PMC8209531; doi:10.2196/27590)

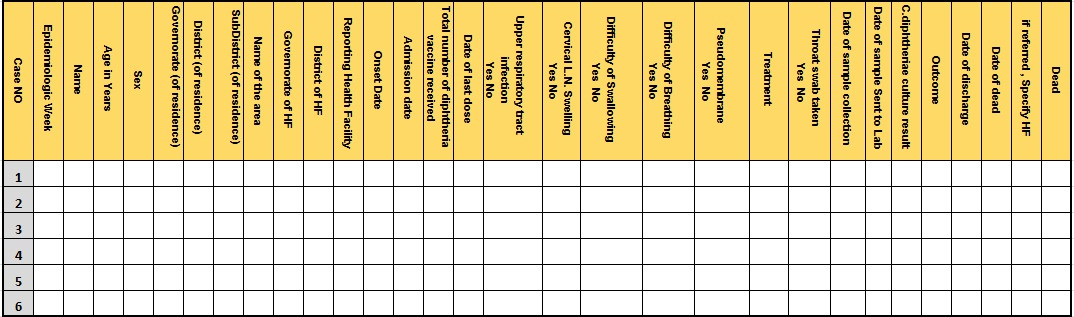

Supplement: Multimedia Appendix 1 [file publichealth_v7i6e27590_app1.png]
